# The neuropathic phenotype of the K/BxN transgenic mouse with spontaneous arthritis: pain, nerve sprouting and joint remodeling

**DOI:** 10.1038/s41598-020-72441-5

**Published:** 2020-09-24

**Authors:** Gilson Gonçalves dos Santos, Juan Miguel Jimenéz-Andrade, Sarah A. Woller, Enriqueta Muñoz-Islas, Martha Beatriz Ramírez-Rosas, Nobuko Ohashi, Glaucilene Ferreira Catroli, Yuya Fujita, Tony L. Yaksh, Maripat Corr

**Affiliations:** 1 Department of Anesthesiology, University of California, San Diego, La Jolla, CA USA; 2 Division of Rheumatology, Allergy and Immunology, University of California, San Diego, 9500 Gilman Dr. MC 0656, La Jolla, CA 92093-0656 USA; 3 Unidad Académica Multidisciplinaria Reynosa-Aztlán, Universidad Autónoma de Tamaulipas, Reynosa, Tamaulipas Mexico; 4 National Institute of Neurological Disorders and Stroke, National Institutes of Health, Rockville, MD 20852 USA

**Keywords:** Rheumatology, Neuroscience, Sensory processing

## Abstract

The adult K/BxN transgenic mouse develops spontaneous autoimmune arthritis with joint remodeling and profound bone loss. We report that both males and females display a severe sustained tactile allodynia which is reduced by gabapentin but not the potent cyclooxygenase inhibitor ketorolac. In dorsal horn, males and females show increased GFAP^+^ astrocytic cells; however, only males demonstrate an increase in Iba1^+^ microglia. In dorsal root ganglia (DRG), there is an increase in CGRP^+^, TH^+^, and Iba1^+^ (macrophage) labeling, but no increase in ATF3^+^ cells. At the ankle there is increased CGRP^+^, TH^+^, and GAP-43^+^ fiber synovial innervation. Thus, based on the changes in dorsal horn, DRG and peripheral innervation, we suggest that the adult K/BxN transgenic arthritic mice display a neuropathic phenotype, an assertion consistent with the analgesic pharmacology seen in this animal. These results indicate the relevance of this model to our understanding of the nociceptive processing which underlies the chronic pain state that evolves secondary to persistent joint inflammation.

## Introduction

Rheumatoid arthritis (RA) is characterized by chronic and symmetric inflammation of synovial joints leading to joint destruction, chronic pain, loss of function, and disability^1–3^. RA may also result in peri-articular bone erosions, and generalized osteoporosis with significant loss of bone mineral density (BMD)^4–6^. Current therapeutics, notably the biologics, have resulted in a significant improvement in the ability to manage the arthritic state^7–9^, but in spite of this efficacy in addressing the evolving joint dysfunction can, paradoxically, leave the associated pain unmanaged^10–14^. It is commonly thought that arthritic pain is mediated by inflammation or tissue damage, leading to a peripheral and central sensitization. Yet, the severity of the pain and the restricted efficacy of many anti-inflammatory agents often does not correlate well with these factors^15^. In fact, patients with RA frequently continue to report pain despite the control or resolution of clinical signs of arthritis^16^.

To better understand the complex mechanisms driving RA associated pain different animal models have been used that are largely induced and not spontaneously occurring^17–20^. Here we examine the K/BxN model^21^ where mice express a transgenic T cell receptor, KRN, that drives the development of autoantibody formation to glucose 6 phosphate isomerase^22^. This autoantibody mediates the development of a robust autoimmune arthritis within a month after weaning in both male and female mice^21,23^. The structural characteristics of this arthritis display a phenotype similar to that found in human RA joints, including inflammatory infiltrates and articular erosions. Although the physical changes in bone and joint of the K/BxN mouse have been well described, the associated pain or the effects upon nociceptive afferent signaling have not been well characterized. Here we characterize in males and females the severity of arthritis, changes in bone parameters from the onset of mechanical allodynia. Further our work and that of others has suggested the possibility that chronic inflammation may lead to changes in the innervation of the joint and activation of signaling in the dorsal horn and dorsal root ganglion (DRG) consistent with a neuropathic phenotype^17,19,20^. These changes together with analgesic pharmacology support the hypothesis that the K/BxN mouse displays a neuropathic phenotype which may be superimposed on the chronic inflammatory state.

## Results

### Adult male and female K/BxN mice display severe erosive arthritis

**Figure 1:**
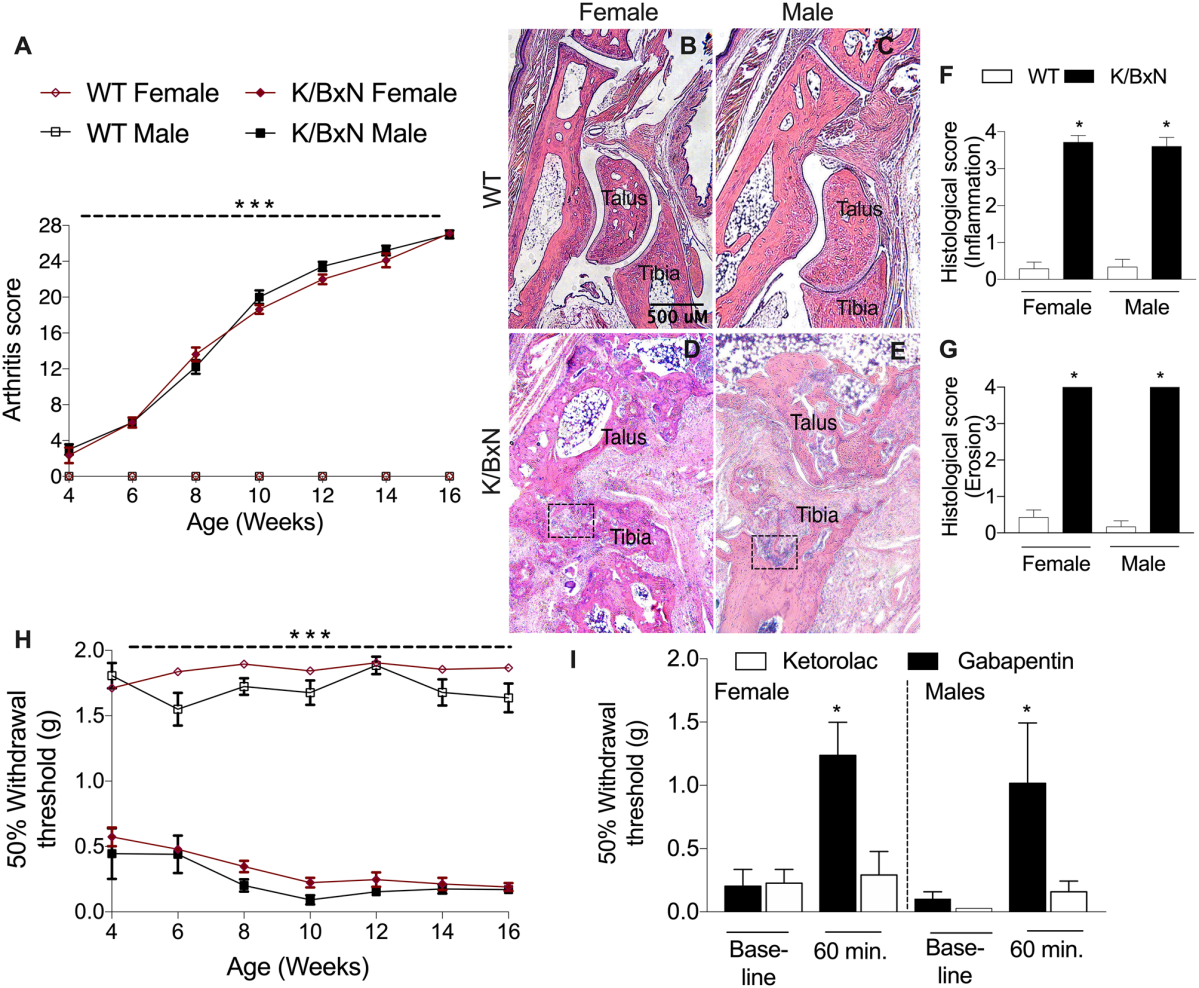
Male and female K/BxN mice develop destructive arthritis and mechanical allodynia. Male and female WT and K/BxN mice (n = 9/group) were followed from 4 to 16 weeks of age scored for arthritis by visual inspection **(A)**. Groups of mice (n = 5–7/group) were sacrificed at 16 weeks and the ankles were histologically examined. Both male and female K/BxN mice had severe bone erosion and cartilage loss **(B–G)**. The same mice in **(A)** were assessed for tactile allodynia by von Frey testing **(H)**. Groups of 16-week-old mice (n = 6/group) were tested for their responses to ketorolac or gabapentin by withdrawal threshold 60 min after drug delivery **(I)**. Data are shown as mean ± SEM. *p < 0.01, ***p < 0.001 by strain two-way ANOVA for repeated measures and Bonferroni post hoc test.

We scored arthritis and measured mechanical withdrawal thresholds every 2 weeks after weaning up to an age of 16 weeks. All transgenic mice showed steadily increasing arthritis scores [F(2,127) = 221, *p* = 0.0001, two-way ANOVA; Fig. 1A]. At sixteen weeks male and female wild type and K/BxN mice were sacrificed and the hind limbs harvested and one limb was decalcified, and prepared for histology. The K/BxN mice had severe damage to the tibial/talar joint (Fig. 1B–E). There were significant erosions and in several cases the ankle bones were fused in the K/BxN transgenic mice. Histologically there were significant differences in the inflammation [F(4,25) = 19.73 p = 0.0002, Fig. 1F] and erosion [F(4,25) = 21.63 p < 0.0001, Fig. 1G] scores between strains but not between sexes.

### Male and female arthritic K/BxN mice develop mechanical allodynia that responds to gabapentin but not ketorolac

K/BxN transgenic mice developed tactile allodynia by age four weeks [F(3,156) = 853, *p* = 0.0001, two-way ANOVA] that persisted up to age 16 weeks compared to wild type mice (Fig. 1H). There were no significant differences between sexes of each strain in mechanical withdrawal thresholds (Fig. 1H). Groups of 16-week-old mice were then tested for their responses to a nonsteroidal anti-inflammatory drug (NSAID), ketorolac given at a dose shown to robustly reduce inflammation and hyperalgesia in models of inflammation ^24^, or gabapentin at doses which were efficacious in models of nerve injury and facilitated processing ^25^ (Fig. 1I). Although both sexes of K/BxN mice had ongoing paw swelling, they displayed minimal if any response to ketorolac [F(7,27) = 12.72, *p* = 0.89, one-way ANOVA]. Instead, both sexes of K/BxN mice responded robustly to gabapentin injection [F(7,27) = 16.47, *p* = 0.001, one-way ANOVA].

### Male and female K/BxN mice demonstrate trabecular bone loss

**Figure 2:**
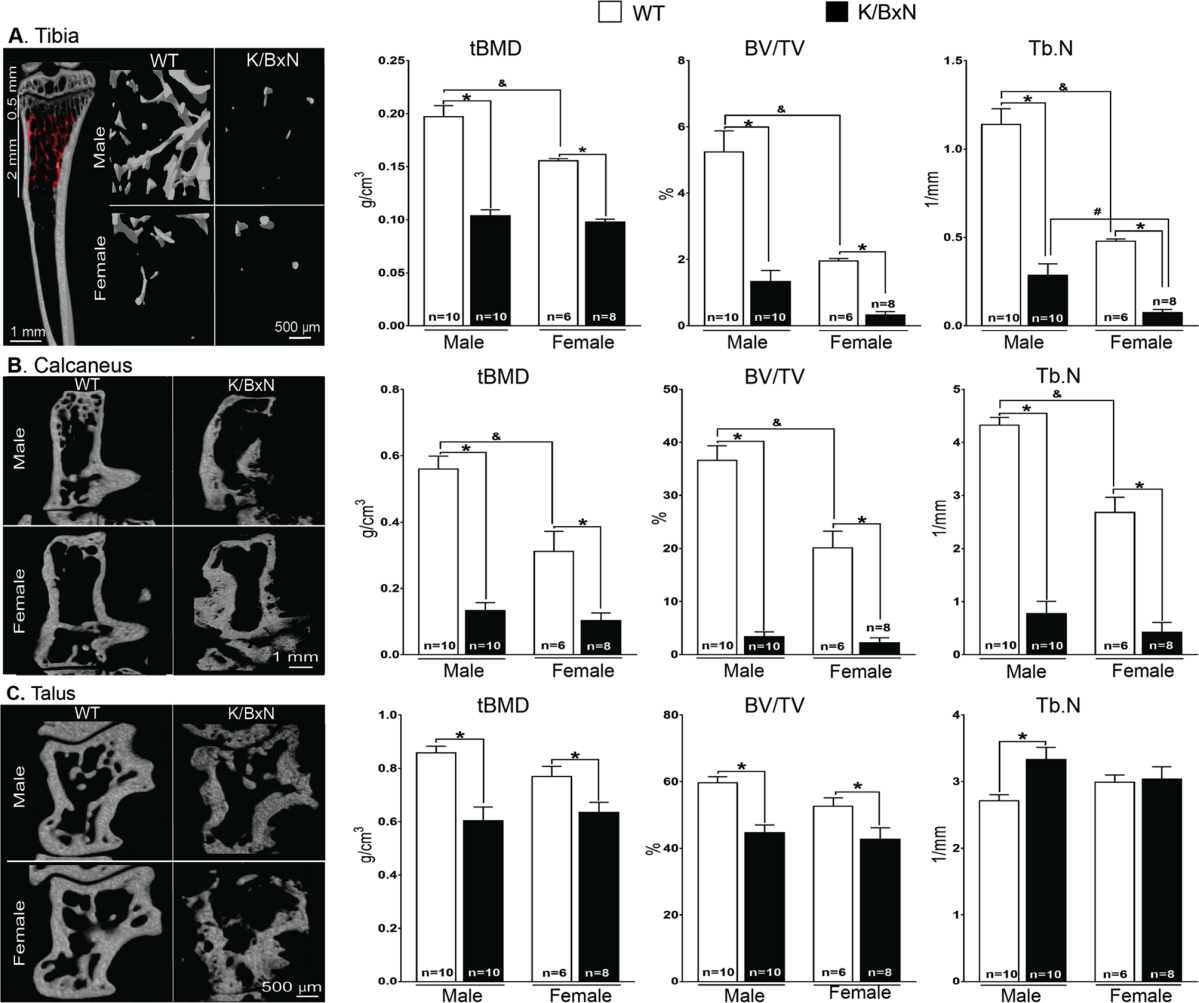
Differences in trabecular bone in WT and K/BxN mice. Representative images of tridimensional reconstructions at different sites and quantitative analyses of trabecular bone parameters **(A–C)**. MicroCT analysis was performed on the tibiae of 16-week-old mice. Female and male K/BxN mice show a decrease of BMD, BV/TV and Tb.N compared with wild type (WT) in the tibiae **(A)**, calcanea **(B)** and tali **(C)**, both in males and females, except for Tb.N of talus in females **(C)**. Data are shown as mean $\pm$ SEM of 6–9 mice/group. *p < 0.05 by strain, ^&^*p* < 0.05 by sex by two-way ANOVA and Bonferroni post hoc test.

The hind limbs were also analyzed by microCT (Fig. 2 and supplemental Table 1 for specific statistical analyses). We measured trabecular bone mineral density (tBMD), bone volume (BV/TV) and trabecular number (Tb.N) in the tibia (Fig. 2A), calcanea (Fig. 2B) and tali (Fig. 2C). There were significant differences in bone structure between sex and strain (Fig. 2 and supplemental Table 1 for specific statistical analyses).

Between strains (WT versus K/BxN) there were significant differences in tBMD, BV/TV and Tb.N for both males and females in the tibia (Fig. 2A), calcanea (Fig. 2B), and tali (Fig. 2C) (*p* < 0.001, two-way ANOVA and Bonferroni post hoc test; Supplemental Table 1) with the exception of Tb.N in the tali of females (Fig. 2C). Similarly, there were significant differences between WT male and female mice in tBMD, BV/TV, and Tb.N of the tibia (Fig. 2A) and calcanea (Fig. 2B) (*p* < 0.001, two-way ANOVA and Bonferroni post hoc test; Supplemental Table 2). However, in the K/BxN mice the only significant sex difference was in Tb.N at the trabecular tibia [F(1,30), *p* = 0.015, two-way ANOVA and Bonferroni post hoc test].

### Differences in dorsal horn glial activation in K/BxN male and female mice

**Figure 3:**
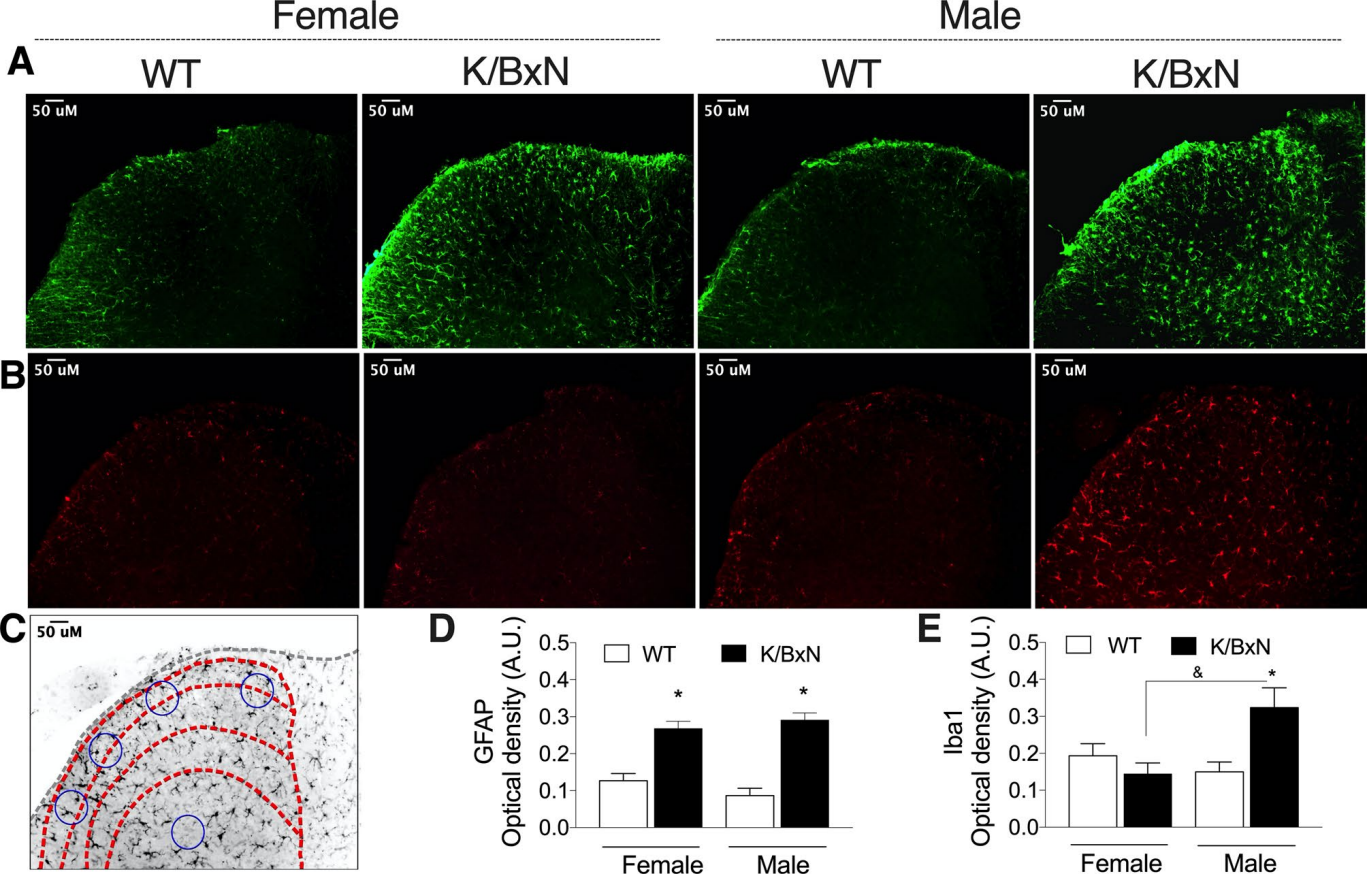
K/BxN male and female spinal cord glial activation. Spinal cords were harvested from 16-week-old male and female WT and K/BxN mice (n = 6–9 mice /group) and the lumber regions were incubated with antibodies against GFAP **(A)** and Iba1 **(B)**. Representative staining images which were used for the Iba1 and GFAP quantification are shown (bar is 50 µm). **(C)** Schematic of Rexed’s laminae I–II (in red) and four circular (blue) areas where the optical density (OD) was analyzed. The OD of an area (blue circle) in the deep region of the same slice was subtracted as the background control. **(D)** GFAP immunofluorescence was increased in both the male and female mice compared to WT. **(E)** Iba1 staining was increased in the male K/BxN mice compared to WT. *p < 0.05 strain, ^&^*p* < 0.05 by sex one-way ANOVA with Bonferroni post hoc test data are represented as mean ± SEM.

We next examined the spinal cords of WT and arthritic K/BxN male and female mice for glial activation by immuno-reactivity in laminae I–III for GFAP (astrocytes; Fig. 3A) and Iba-1 (microglia; Fig. 3B). Quantification of GFAP staining is elevated in both the female and male K/BxN mice at week 16 [F(3,25) = 16.98, *p* = 0.0002, one-way ANOVA; Fig. 3C]. However, Iba1 staining was increased only in the K/BxN males [F(3.25) = 5.81, *p* = 0.0001, one-way ANOVA; Fig. 3D].

### Increased tyrosine hydroxylase and calcitonin gene related peptide in the dorsal root ganglion of K/BxN males and females

**Figure 4:**
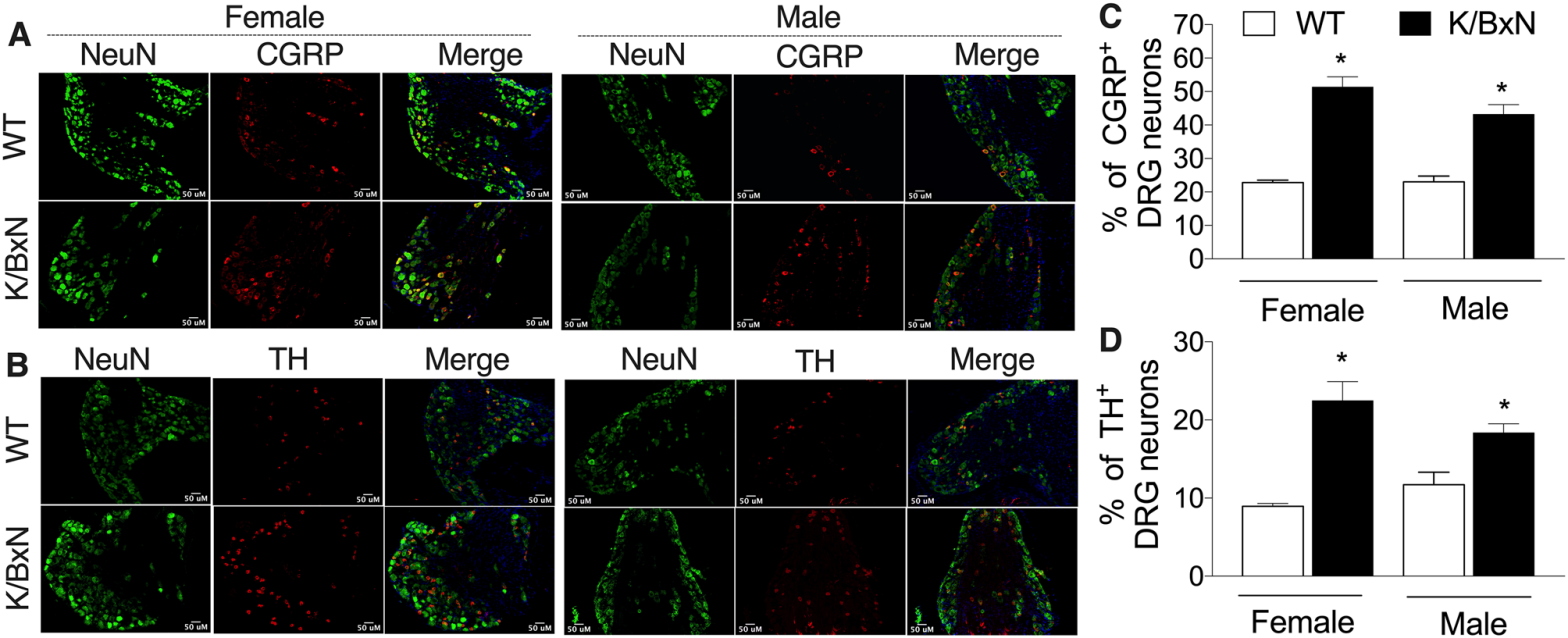
K/BxN male and female have increased CGRP^+^ and TH^+^ neurons in the DRG. The L4 and L5 DRG were harvested from 16-week-old male and female WT and K/BxN mice (n = 7–8 mice /group) and stained for DAPI (blue), NeuN (green), TH (red) and CGRP (red). Representative staining images are shown (bar is 50 µM), which were used for quantification of CGRP **(A)** and TH **(B)**. The mean percentages ± SEM of NeuN positive cells that also stained for CGRP **(C)** and TH **(D)** are shown. *p < 0.05; one-way ANOVA with Bonferroni post hoc test. There were no differences between sexes of the same strain (*p* > 0.05).

The L4-5 DRG were removed and stained for DAPI, NeuN, tyrosine hydroxylase (TH, a marker of sympathetic nerve fibers) and calcitonin gene related peptide (CGRP, a marker for peptide-rich C and Aδ nerve fibers; Fig. 4). Quantification of staining showed that there was significant increase in the number of TH^+^ neurons [F(3,22) = 2.42, *p* = 0.0001, one-way ANOVA, Fig. 4B,D] and CGRP^+^ neurons [F(3,16) = 2.42, *p* = 0.0001, one-way ANOVA, Fig. 4A,C] in the DRGs of K/BxN mice in both sexes.

### K/BxN males and females increase Iba1, but not ATF3 activation in the DRG

**Figure 5:**
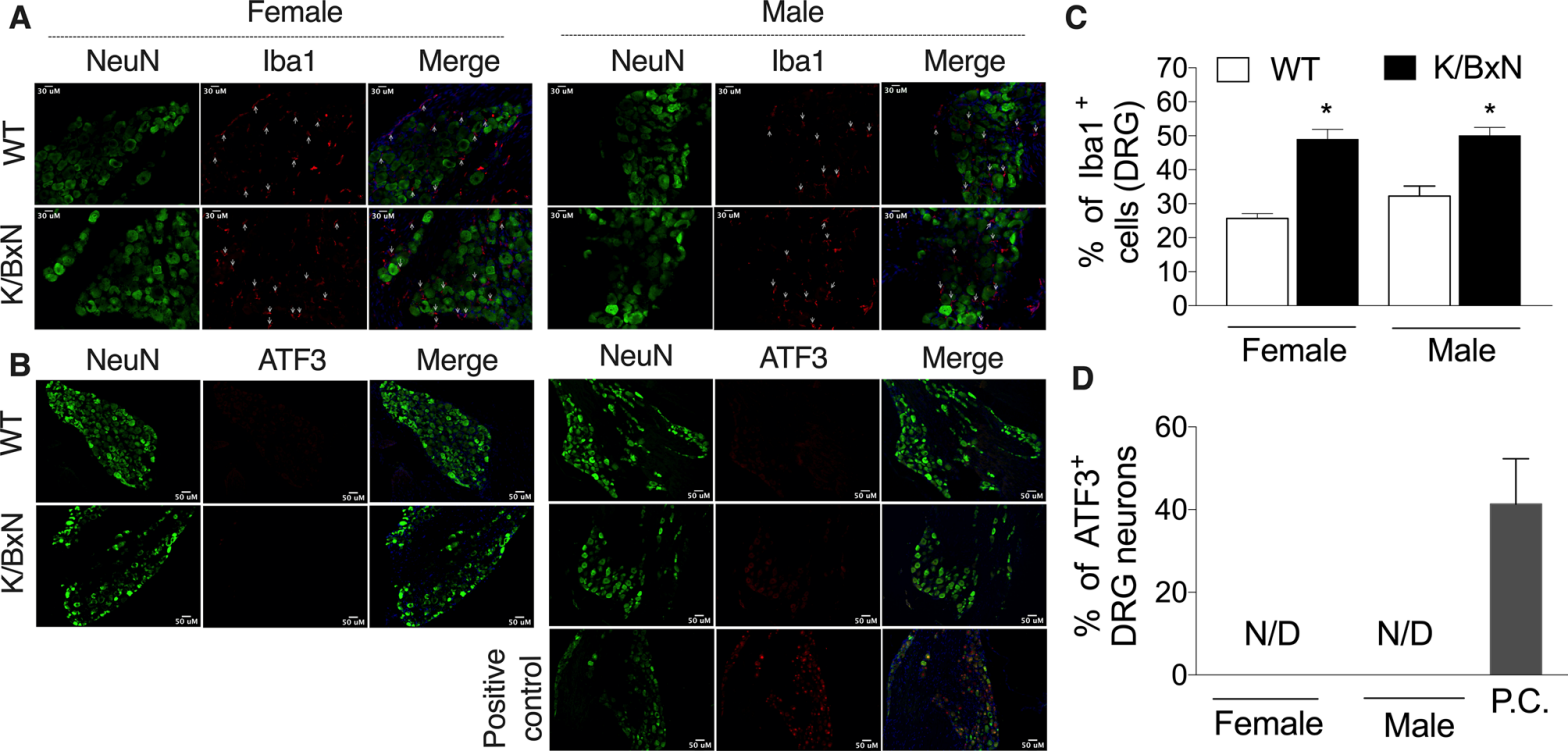
Arthritis is followed by increase of macrophage (Iba1) with lack of ATF3 expressing neurons in the DRG of K/BxN mice. The L4 and L5 DRG were harvested from 16-week-old male and female WT and K/BxN mice (n = 6 mice /group) and stained for DAPI (blue), NeuN (green), Iba1 (red) and ATF3 (red). Representative staining images are shown (bar is 50 µM), which were used for quantification of Iba1 **(A)** and ATF3 **(B)**. The mean percentages ± SEM of NeuN positive cells that also stained for Iba1 **(C)** and ATF3 **(D)** are shown. *p < *0*.05 one-way ANOVA with Bonferroni post hoc test. There were no differences between sexes of the same strain (*p* > 0.05). **(D)** No immunoreactivity for ATF3 was found in the DRG of WT or K/BxN; however, L5 nerve axotomy induced an increase of ATF3 in at least 50% of neurons on the DRG. *p < 0.05 one-way ANOVA with Bonferroni post hoc test. *N.D.* not detected.

The L4-5 DRGs were removed and stained for DAPI, NeuN, Iba1 and activated transcription factor 3 (ATF3, Fig. 5). Quantification of staining showed that there was significant increase in the number of Iba1^+^ cells in the DRG of K/BxN mice in both sexes [F(3,16) = 2.42, *p* = 0.01 one-way ANOVA, Fig. 5A,C]. Strikingly, there was an absence of ATF3^+^ cells in the DRGs in any of the WT or the K/BxN mice of either sex (Fig. 5B,D). DRG sections from a prior L5 nerve axotomy were used as a positive control to verify the function of the ATF3 antibody. There was an increase in the Iba1 staining seen in the same samples as a control for the integrity of the specimen (Fig. 5).

### Increases in sensory and TH^+^ nerve fiber expression and sprouting in K/BxN mice

**Figure 6:**
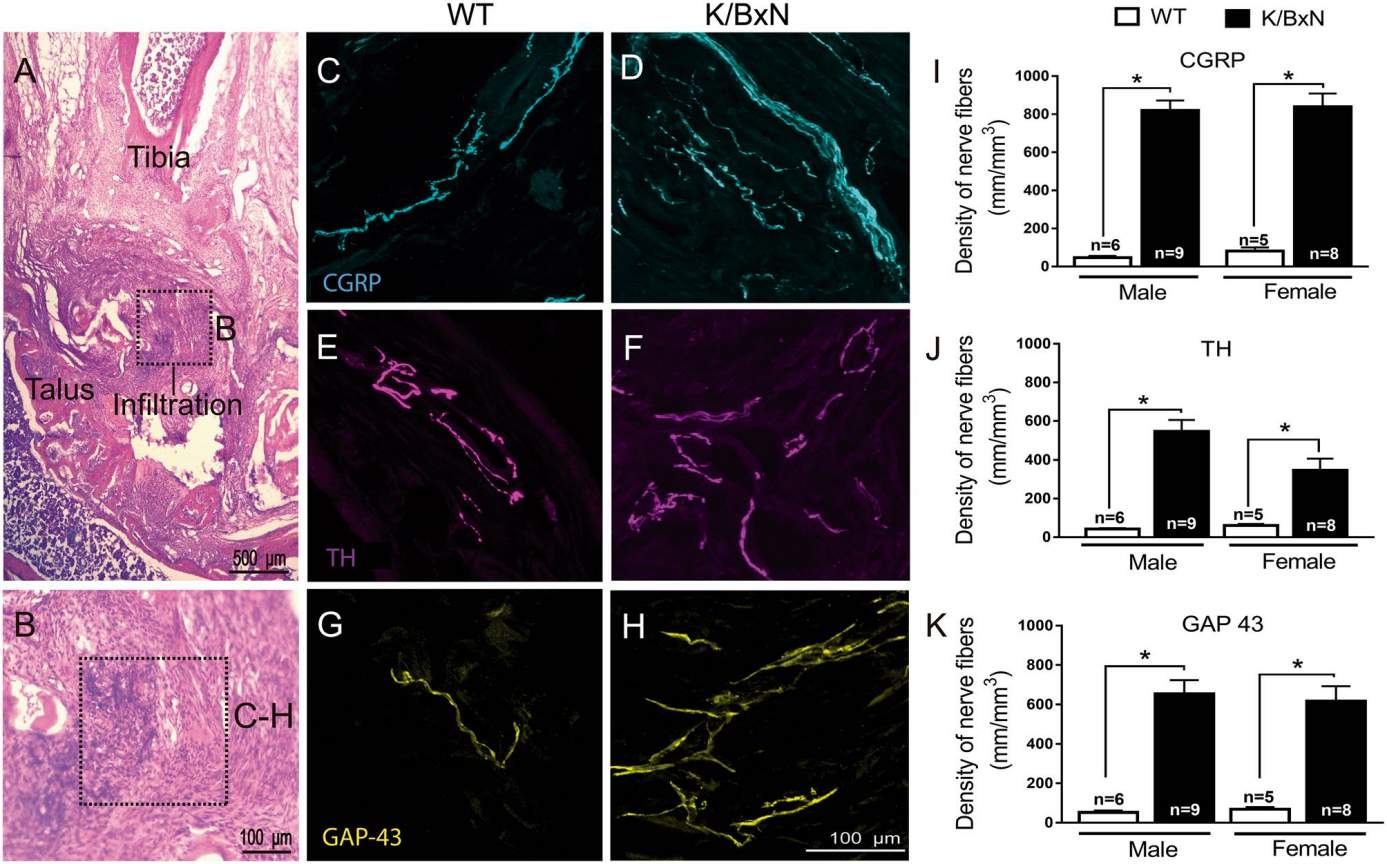
Increase in sensory CGRP^+^, TH^+^ and GAP-43 nerve fibers in arthritic ankles*.* **(A)** Hematoxylin and eosin staining of a K/BxN ankle joint at 16 weeks (bar is 400 µm). The square illustrates the synovial region from which the confocal images were obtained (**B**; bar is 100 µm). Representative confocal images of calcitonin gene-related peptide (CGRP; cyan, **C**, **D**), tyrosine hydroxylase (TH; purple, **E**, **F**) and growth-associated protein (GAP-43, marker of fibers undergo regeneration; yellow, **G**, **H**) in ankle-joint sections (20 μm thick) from WT **(C, E, G)** and K/BxN **(D, F, H)** mice (bar is 100 µm). A significantly greater density of CGRP^+^, TH^+^ and GAP-43^+^ sprouted nerve fibers are observed in the synovia of K/BxN as compared to those observed in WT mice **(I, J, K)**. Bars represent the mean ± SEM of 6–9 mice/group. *p < 0.05 one-way ANOVA with Bonferroni post hoc test.

The ankle joints of 16-week-old K/BxN mice and controls were examined by immunohistochemistry for the quantification of sympathetic and peptidergic nerve fibers in the synovium. First, there was a profound inflammatory infiltrate seen by H&E staining in the joints of the transgenic mice, consistent with prior reports (Fig. 6A,B). In WT mice, a low-level, regular pattern of innervation by CGRP^+^ and TH^+^ nerve fibers was observed in the synovium. In these mice, there was a robust increase in the density of TH^+^ (used as marker for sympathetic nerves)^26,27^, CGRP^+^ (sensory) and GAP-43^+^ fibers (nerve fibers undergoing sprouting) in both the male and female K/BxN mice (Fig. 6C–K). In the transgenic mice, a significant number of CGRP^+^, TH^+^, and GAP-43^+^ nerve fibers had sprouted and had a disorganized appearance, as compared with wild type mice (Fig. 6). Quantitative analysis of the density of nerve fibers (expressed as density of nerve fibers) in the synovial membrane (ankle joint) revealed that the K/BxN group had a significant increase in the density of CGRP^+^ fibers as compared with WT mice in males (819.9 ± 55.6 versus 67.0 ± 16.0 mm/mm^3^; *p* < 0.05) and females (839.3 ± 69.7 versus 79.5 ± 15.0 mm/mm^3^; *p* < 0.05). Likewise, the density of TH^+^ was significantly increased in K/BxN males (546.6 ± 38.7 versus 46.13 ± 7.0 mm/mm^3^; *p* < 0.05) and female (346.6 ± 60.2 versus 60.4 ± 7.4 mm/mm^3^; *p* < 0.05). Finally, the density of nerve fibers undergoing sprouting indicated by GAP-43^+^ staining also increased significantly in K/BxN males (653.8 ± 70.0 versus 76.7 ± 10.1 mm/mm^3^; *p* < 0.05) and female (616.9 ± 76.1 versus 65.8 ± 9.0 mm/mm^3^; *p* < 0.05).

**Figure 7:**
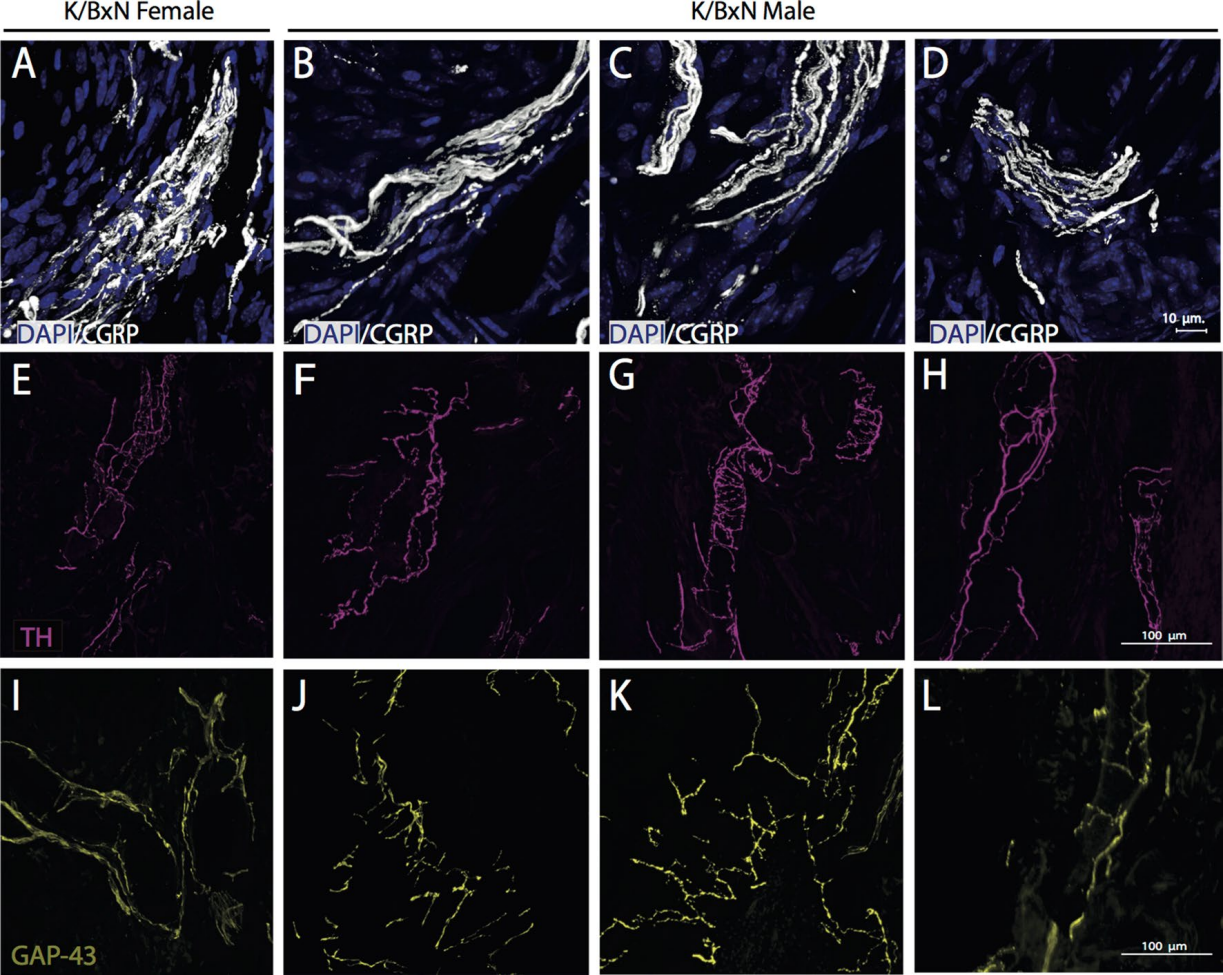
Dense bundles of CGRP^+^ fibers near the ankle/calcaneus of K/BxN mice. Confocal images of dense fibers in female **(A)** and three males **(B–D)** 16-week-old K/BxN mice are shown (bar is 100 µm). DAPI-labeled nuclei (blue; **A–D**), and CGRP (white; **A–D**), TH (purple; **E–H**) and GAP-43 (yellow; **I–L**) immunoreactive nerve fibers in the ankle joint sections show that the densest bundles were formed by CGRP^+^ nerve fibers in the synovia of arthritic mice.

Whereas all K/BxN mice showed significant and robust increases in CGRP^+^, GAP-43^+^, and TH^+^ nerve fibers, indicative of sprouting, CGRP + fibers were structurally prominent in three out of nine male mice and one of eight female mice with at least 10 individual CGRP + axons that were > 20 µM thick (Fig. 7).

## Discussion

In arthritic pathologies, such as RA, joints can remain painful after successful treatment of synovial inflammation. The quality of this pain can have neuropathic features^28,29^. One potential mechanism that may explain the dissociation between disease progression and pain in arthritis is an ectopic sprouting of sensory and sympathetic nerve fibers^18,19,30,31^. Here we examined male and female KRN T cell receptor transgenic mice for the onset of mechanical tactile allodynia and characterized them for central changes in the spinal cord, evidence of nerve injury in the DRG and peripheral sprouting of sympathetic and sensory nerves near the damaged joints. We evaluated both male and female animals, as there are reported differences in pain mechanisms between sexes^32–34^.

In this series of studies, clinical and histologic signs of inflammation did not display strong covariance with withdrawal thresholds. Several elements point to this characteristic as reflecting a neuropathic, as opposed to an inflammatory phenotype:

(i) Ketorolac, a potent cyclooxygenase inhibitor^24^ had a minimal impact on the mechanical allodynia. In contrast, gabapentin, which displays efficacy in neuropathic models through binding to the alpha 2 delta1 subunit of the voltage gated Ca^2+^ channel (reviewed in^35^), was highly effective. Importantly, while only a single dose of each was used, the ketorolac dose was chosen based on its efficacy in reducing inflammatory indices and hyperalgesia/ allodynia otherwise observed in robust models of inflammation^24^. Similarly gabapentin dosing was based on demonstrated efficacy and absence of effects upon spontaneous behavior in murine models of facilitated processing and polyneuropathy^25^. However, we note that there may have been time confounds in our protocol as the drugs were administered serially and not in a cross-over design.

(ii) In the adult K/BxN mice there was a noted sex difference in glial activation markers. Both males and females demonstrated an increase in GFAP but only males demonstrated an increase in Iba1 labeling which indicate activated astrocytes and microglia respectively. Glial activation has been implicated in the development and maintenance of a neuropathic condition^36^. Recent work has shown that microglia (Iba1) are not required for mechanical pain hypersensitivity in female mice. In one study, minocycline did not prevent mechanical allodynia female mice^32^. Moreover, only in male, pain related behavior seems to be driven by P2X4R-induced release of BDNF by microglia^37^.

(iii) Although ATF3 is frequently cited as a marker of nerve injury^38^, no increase in ATF3 staining was seen in the DRGs of these adult mice. We note that in the K/BxN and the collagen antibody-induced arthritis (CAIA) passive transfer models of arthritis, ATF3^+^ staining was demonstrated in the lumbar DRGs after approximately 10–15 days^17,20^. This increase in ATF3 was however typically transient and in the CAIA model the ATF3 staining was markedly diminished at a later time point^20^. Similarly, while nerve ligations also lead to significant increases in DRG-ATF3 expression, this increase secondary to direct nerve injury can disappear by 14 days^39^. Hence, the absence of DRG-ATF3 staining was not inconsistent with a neuropathic phenotype. As the present immunohistochemical studies focused on adults, it is reasonable that we might find such changes during an earlier stage of development in this arthritis phenotype.

(iv) In the K/BxN transgenic mice, CGRP^+^, TH^+^, and GAP-43^+^ nerve fibers were observed to show a significant increase in fiber density and, in contrast to the regular pattern of innervation seen in WT mice, displayed a highly ramified disorganized appearance. CGRP is uniformly considered to reflect small afferent innervation^40^. TH has been extensively used as marker for sympathetic post-ganglionic innervation to study peripheral sprouting in pathological conditions^18,27^. However, we note that TH is also expressed in a subpopulation of small DRG neurons^41^. This raises the question whether the peripheral joint innervation expressing TH reported here represents sensory, post ganglionic sympathetic or both. TH expressing DRG neurons innervating non-visceral tissues seldom co-express CGRP, in contrast to visceral innervation^42^. It thus appears likely that at least some of the upregulated expressing axons represent post ganglionic fibers. Additional work is required to address this possibility.

In all, these findings are consistent with previous studies in humans and rodents indicating that remodeling of sensory and sympathetic nerve fibers can be associated with musculoskeletal pain^18,19,30,31,43,44^. Sprouting of afferents and TH^+^ fibers along with the formation of neuroma like structures can lead to spontaneous discharges^45–47^. This property of initiating ectopic/spontaneous discharges can explain in part components of the chronic arthritis pain, which is refractory to anti-inflammatory interventions. It should be noted that this sprouting may be a generalized response to chronic joint inflammation. Significant increases in CGRP, GAP-43 and TH have also been reported after repeated intra articular injections of complete Freund’s adjuvant^18,31^. Hence, the ectopic sprouting of sensory and sympathetic nerve fibers reported in separate models of chronic murine arthritis, suggests a common mechanism may be involved in the generation and maintenance of the arthritic pain in the chronic animals. These observations suggest that the transgenic K/BxN mouse provides a specific phenotype for the chronic inflammatory state that may arise from an early onset joint inflammation and suggest that that underlying pain state reflects a polyneuropathic phenotype in both sexes. This distinction may be of particular significance in screening for the development of new analgesic therapeutics for these chronic states given the robust distinction observed here between the actions of a NSAID and a centrally active anti-hyperalgesic, gabapentin.

## Methods

### Mice

Male (n = 12) and female (n = 12) C57BL/6 wild type (WT) mice were purchased from Harlan (Indianapolis, IN), and were given at least 48 h to acclimate to the vivarium before use. Other mice were obtained as breeder pairs and were bred/maintained under standard conditions in a University of California, San Diego Animal Facility that is accredited by the American Association for Accreditation of Laboratory Animal Care. KRN T cell receptor transgenic mice were a gift from Drs. D. Mathis and C. Benoist (Harvard Medical School, Boston, MA and Institut de Génétique et de Biologie Moléculaire et Cellulaire, Strasbourg, France) ^21^. These mice were maintained on a C57BL/6 background (K/B). Arthritic mice were obtained by crossing K/B with NOD/Lt (N) mice (K/BxN). Twelve female and twelve male transgenic mice were used. All animal experiments were conducted according to protocols approved by the Institutional Animal Care and Use Committee of the University of California, San Diego. Mice were housed up to 4 per standard cage at room temperature and maintained on a 12:12 h light: dark cycle. All behavioral testing was performed during the light cycle. Both food and water were available ad libitum.

### Arthritis scoring

The development of joint inflammation in the paws was evaluated by visual inspection and rated on a scale of 1 to 28 where one point was given for each swollen digit and two points for each swollen ankle or wrist (modified from a prior report^48^).

### Von Frey behavioral testing

Mechanical withdrawal thresholds were assessed every other week using the up-down method^49^. Briefly, animals were placed in clear, plastic, wire mesh-bottomed cages for 45-min prior to the initiation of testing. Tactile thresholds were measured with a series of calibrated von Frey filaments (Seemes Weinstein von Frey anesthesiometer; Stoelting Co., Wood Dale, IL, USA) ranging from 2.44–4.31 (0.02–2.00 g). Mechanical values for the left and right hind paws were measured and the 50% probability of withdrawal threshold was calculated ^49^. Thresholds for the two hind paws were averaged to produce a single data point per day of measurement.

### Drugs and drug delivery

To define the analgesic pharmacology of the K/BxN allodynic state, groups of 16-week-old K/BxN transgenic arthritic mice (n = 6/sex) received a single i.p. injection of gabapentin (100 mg/kg; Pfizer), or the NSAID, ketorolac (7.5 mg/kg; Sigma). Mice were measured for changes in tactile allodynia 1 h following treatment. Drugs were allowed to wash out for 48 h before the same procedure was repeated with a different drug. Gabapentin^50,51^, and ketorolac^24^ were dissolved in normal saline, dosages were based on previous reports demonstrating anti-allodynic efficacy, absent motor or general behavioral effects, in murine models of mono / poly and inflammatory neuropathy, respectively.

### Histology and immunohistochemistry

At sixteen weeks, mice were deeply anesthetized with Beuthanasia-D and perfused intracardially with 0.9% saline followed by 4% paraformaldehyde. The spinal cords and lumbar DRGs were removed, post-fixed, and cryoprotected in 30% sucrose. The ankle in the mouse is innervated with varying contributions by levels L3–L5 depending on the strain, with C57BL/6 mice trending toward L4–L5^52^. Lumbar sections (L4–L5) of the spinal cord were cut on a microtome (30 μm) as free-floating sections. Tissue sections were incubated with anti-GFAP (glial fibrillary acidic protein) antibody (1:1,000 Sigma, St. Louis MO) or anti-Iba1 (Ionized calcium binding adaptor molecule (1) antibody (1:1,000 Wako, Richmond, VA), washed, and then incubated with secondary antibodies conjugated with fluoro-Alexa-488 and Alexa-594 (1:500, Molecular Probes, Eugene, OR). Images were captured by Olympus BX51 system and quantified by a blinded investigator using ImageJ (National Institutes of Health). As described^53,54^ microglia (Iba1) and astrocyte (GFAP) labeling was quantified by measuring the fluorescence intensity converted to optical density (OD) in arbitrary units in laminae I and II of the dorsal horn of the spinal cord in ImageJ. Initially each picture was converted to 8-bit gray scale. We then calibrated each section using the “uncalibrated OD” function. Four circular regions of interest (diameter = 88 µM) were placed in laminae I and II. The OD from an equivalent area in a deep layer of the same slice was subtracted as the background from the average reading.

The L4–L5 DRGs were cut in transverse sections (14 μm) and mounted on glass slides. DRGs were incubated with anti-NeuN (neuronal nuclei) biotinylated (1:1,000, Chemicon), anti-ATF3 (1:1,000, Santa Cruz), anti-Iba1 antibody (1:1,000 Wako, Richmond, VA), anti-TH (1:1,000, Millipore), anti-CGRP (1:1,000, Sigma) and nuclear stain 4′,6-diamidino-2-phenylindole (DAPI). Binding sites were visualized with secondary antibodies conjugated with fluoro-Alexa-488. The total number of immunoreactive cells were counted in each section (four sections per animal). The number of NeuN positive nuclei per section was used as the denominator for the percentage of neurons that were CGRP^+^, TH^+^ or ATF3^+^. At least six animals are included for each group.

To analyze periarticular innervation, once hind extremities were harvested, they were post-fixed for 24 h and placed in 0.01 M PBS (pH = 7.4) at 4 °C until their micro computed tomography (microCT) analysis. Once microCT was finalized, samples were placed in a 10% EDTA solution for two weeks for decalcification. The degree of decalcification was monitored at regular intervals by plain radiography. Then, decalcified bones were cryoprotected in a 30% sucrose solution at 4 °C for further processing by immunohistochemistry. The femora were cut into serial cross sections (20 μm thick) with a cryostat (MTC, SLEE, Mainz, Germany)^31,55–57^. Frozen ankle joint sections were incubated with antibody against calcitonin gene-related peptide (CGRP, polyclonal rabbit anti-rat CGRP; 1:4,000; Sigma) to label primary afferent sensory nerve fibers. Sympathetic nerve fibers were labeled with antibody against tyrosine hydroxylase (TH, polyclonal rabbit anti-rat TH, 1:1,000; EMD Millipore). Sprouted nerve fibers were labeled with an antibody against growth-associated protein-43 (GAP-43, rabbit anti-GAP-43, 1:1,000; EMD Millipore). Nuclear stain 4′,6-diamidino-2-phenylindole (DAPI) was used to visualize all cell bodies.

For quantification of nerve fiber density, approximately 15 separate, 20-μm-thick frozen sections were obtained from the ankle joint of each mouse. For each given marker, three sections from one ankle joint were initially scanned at low magnification (× 10) to identify the areas with the highest density of nerve fibers thorough an epifluorescence microscope (Axio Scope.A1, Carl Zeiss, Jena, Germany). These areas in the synovium were defined as "hot spots"^31^. One image per section was acquired within the medial synovial hot spot. Then, an image was obtained for each section in this area (separated by at least 100 μm) at 40 × magnification with an epifluorescence microscope. The images were analyzed using ImageJ software (National Institutes of Health) and nerve fibers were manually traced by a blinded investigator, using the freehand line tool, to determine the total length of nerve fibers. Data from at least three sections per ankle joint were recorded and averaged. Total volume of the synovium (naïve and inflamed) was calculated by tracing the area of the synovium and multiplying this area by the thickness of the section (20 μm). Data are expressed as total length of nerve fibers per volume of synovium (mm/mm^3^)^18,26,31^. Representative images from WT and transgenic mice for each marker were captured with a Carl Zeiss scanning confocal laser microscope (Model LSM 800, Jena Germany). Confocal images used for illustration were assembled and labeled using Adobe Illustrator software. At least two sections (10-μm-thick) from each mouse, were stained with hematoxylin and eosin (H&E) to identify the morphology and integrity of the ankle joints. Representative images from H&E-stained sections were captured with a light microscope Axio Scope.A1, Carl Zeiss, Jena, Germany). One section per mouse was scored in a blinded manner for inflammation and erosion using a semi-quantitative scale (0–4) as previously described^58^.

### Microcomputed tomography

To assess changes in bone parameters, microCT analysis was performed in the trabecular tibia, calcaneus, and talus using a desktop microCT system (Skyscan 1272; Bruker, Belgium). The scanning parameters were: 10 µm voxel size, at 60 kVp and 166 µA with an integration time of 627 ms, according to the guidelines for microCT analysis of rodent bone structure^59^. All the scanner images were reconstructed using NRecon Software (Bruker, Belgium). The trabecular region of interest (ROI) at distal femur and proximal tibia was evaluated by selecting 2 mm in the vertical axis, subsequent to 0.5 mm from the growth plate (reference point). For the calcaneus, analysis ROI was selected using a 0.5 mm^2^ cylinder that was positioned underneath the growth plate of the calcaneus bone and finally for the talus analysis ROI was selectively bordered manually. The CT analyzer program (Bruker, Belgium) was used to determine trabecular bone parameters; an automatic segmentation algorithm (CT analyzer) was applied in order to isolate the trabecular bone from the cortical bone. The parameters used for the trabecular bone were: trabecular bone mineral density (BMD), trabecular bone volume rate (BV/TV), and trabecular number (Tb.N). Finally, hydroxyapatite calibration phantoms (250 and 750 mg/cm^3^) were used to calibrate trabecular bone density values (BMD).

### Statistics

Results are represented as means ± SEM. Statistical analysis was performed using GraphPad Prism (version 8.3.0; GraphPad Software, San Diego, CA, USA). Minimum group sizes were predicated on extensive previous work ^17,60,61^ and demonstrated as appropriate for establishing as statistically significant (p < 0.05) changes in paw diameter and tactile thresholds that were considered to be biologically and behaviorally relevant, as compared to vehicle or baseline controls. This group size was also considered to be relevant for demonstrating joint sprouting and arthritic bone remodeling based on our previous work^62^. For multiple comparisons one or two-way ANOVA was used with Bonferroni post hoc test. In all cases, *p* < 0.05 was considered significant.

## Supplementary information

Supplementary Information.

## Data Availability

Authors can confirm that all relevant data are included in the article and/or its supplementary information file.
