# Supplementary material for: The neuropathic phenotype of the K/BxN transgenic mouse with spontaneous arthritis: pain, nerve sprouting and joint remodeling
Source: Sci Rep. 2020 Sep 24;10:15596. doi: 10.1038/s41598-020-72441-5 (PMC7515905; doi:10.1038/s41598-020-72441-5)
Supplement: Supplementary file 1 — Supplementary Information. [file 41598_2020_72441_MOESM1_ESM.pdf]

# The neuropathic phenotype of the K/BxN transgenic mouse with spontaneous arthritis: Pain, nerve sprouting and joint remodeling

Gilson Gonçalves dos Santos, Juan Miguel Jimenéz-Andrade, Sarah A. Woller, Enriqueta Muñoz-Islas, Martha Beatriz Ramírez-Rosas, Nobuko Ohashi, Glaucilene Ferreira Catroli, Yuya Fujita, Tony L. Yaksh, and Maripat Corr

Supplemental Table 1. Analyses of microCt data by strain*

| | WT x K/BxN (male) | | | | WT x K/BxN (female) | | | |
|---|---|---|---|---|---|---|---|---|
| | Femora | Tibia | Calcanea | Tali | Femora | Tibia | Calcanea | Tali |
| **tBMD** | F(1,30)=58.79, p<0.001 | F(1,30)=105.82, p<0.001 | F(1,30)=75.14, p<0.001 | F(1,30)=22.03, p<0.001 | F(1,30)=58.79, p<0.001 | F(1,30)=105.82, p<0.001 | F(1,30)=75.14, p<0.001 | F(1,30)=22.03, p<0.001 |
| **BV/TV** | F(1,30)=27.78, p<0.001 | F(1,30)=39.47, p<0.001 | F(1,30)=143.36, p<0.001 | F(1,30)=22.67, p<0.001 | F(1,30)=27.78, p<0.001 | F(1,30)=39.47, p<0.001 | F(1,30)=143.36, p<0.001 | F(1,30)= 22.67, p<0.001 |
| **Tb.N** | F(1,30)=55.51, p<0.001 | F(1,30)=85.30, p<0.001 | F(1,30)=184.35, p<0.001 | F(1,30)=4.57, p=0.041 | F(1,30)=55.51, p<0.001 | F(1,30)=85.30, p<0.001 | F(1,30)=184.35, p<0.001 | F(1,30)=184.35, p=0.840 |

*Two-way ANOVA with Bonferroni post hoc correction: p <0.05 in black and p >0.05 in blue

Supplemental Table 2. Analyses of microCt data by sex&

| | Male x Female (WT) | | | | Male x Female (K/BXN) | | | |
|---|---|---|---|---|---|---|---|---|
| | Femora | Tibia | Calcanea | Tali | Femora | Tibia | Calcanea | Tali |
| **tBMD** | F(1,30)=12.04, p=0.002 | F(1,30)=10.48, p=0.003 | F(1,30)=14.40, p<0.001 | F(1,30)=0.493, p=0.156 | F(1,30)=12.04, p=0.327 | F(1,30)=10.48, p=0.556 | F(1,30)=14.40, p=0.548 | F(1,30)=0.493, p=0.588 |
| **BV/TV** | F(1,30)=16.39, p<0.001 | F(1,30)=23.89, p<0.001 | F(1,30)=17.07, p<0.001 | F(1,30)=2.925, p=0.078 | F(1,30)=16.39, p=0.286 | F(1,30)=23.89, p=0.100 | F(1,30)=17.07, p=0.686 | F(1,30)=2.925, p=0.593 |
| **Tb.N** | F(1,30)=21.29, p<0.001 | F(1,30)= 41.06, p<0.001 | F(1,30)=21.87, p<0.001 | F(1,30)=0.0027, p= 0.239 | F(1,30)=21.29, p=0.190 | F(1,30)= 41.06, p<0.001 | F(1,30)=21.87, p=0.233 | F(1,30)=0.0027, p= 0.176 |

&Two-way ANOVA with Bonferroni post hoc correction: p <0.05 in black and p >0.05 in blue
